# Multifaceted Marine Peptides and Their Therapeutic Potential

**DOI:** 10.3390/md23070288

**Published:** 2025-07-15

**Authors:** Svetlana V. Guryanova, Tatiana V. Ovchinnikova

**Affiliations:** 1 M.M. Shemyakin and Yu.A. Ovchinnikov Institute of Bioorganic Chemistry, Russian Academy of Sciences, 117997 Moscow, Russia; svgur@ibch.ru; 2 Medical Institute, Peoples’ Friendship University of Russia, 117198 Moscow, Russia; 3 Moscow Center for Advanced Studies, 123592 Moscow, Russia; 4 Department of Biotechnology, I.M. Sechenov First Moscow State Medical University, 119991 Moscow, Russia

**Keywords:** antimicrobial peptides, host defense peptides, innate immunity, antibacterial, antibiofilm, antifungal, antiviral, antiparasitic, anticancer, immunomodulatory, antihypertensive, antinociceptive, antioxidant, antiaging, antiphotoaging, antithrombotic, wound healing

## Abstract

Marine peptides, derived from a great number of aquatic organisms, exhibit a broad spectrum of biological activities that hold a significant therapeutic potential. This article reviews the multifaceted roles of marine peptides, focusing on their antibacterial, antibiofilm, antifungal, antiviral, antiparasitic, cytotoxic, anticancer, immunomodulatory, chemotactic, opsonizing, anti-inflammatory, antiaging, skin-protective, and wound-healing properties. By elucidating mechanisms of their action and highlighting key research findings, this review aims to provide a comprehensive understanding of possible therapeutic applications of marine peptides, underscoring their importance in developing novel drugs as well as in cosmetology, food industry, aquatic and agriculture biotechnology. Further investigations are essential to harness their therapeutic potential and should focus on detailed mechanism studies, large-scale production, and clinical evaluations with a view to confirm their efficacy and safety and translate these findings into practical applications. It is also important to investigate the potential synergistic effects of marine peptide combinations with existing medicines to enhance their efficacy. Challenges include the sustainable sourcing of marine peptides, and therefore an environmental impact of harvesting marine organisms must be considered as well.

## 1. Introduction

Marine organisms are rich sources of bioactive compounds including peptides which garnered attention for their diverse possible use as therapeutic agents. The potential of marine organisms as sources of biologically active substances and nutritional resources is significant and is expanding every year with the discovery of new species [1,2,3]. More than 2300 new species of marine organisms are registered every year [4]. Moreover, over the past seven decades, the number of new species of marine organisms discovered has exceeded the number of terrestrial species [5]. To date, more than 15,000 molecules isolated from marine organisms have been shown to possess pharmacological potential, and the biomass of phytoplankton is estimated to exceed that of terrestrial organisms [6].

The huge diversity of bioactive peptides in marine organisms is explained by the fact that 1 mL of seawater contains about 1 million bacteria from which marine organisms must be reliably protected [7]. On the other hand, such an abundance of bacteria can also be a source of bioactive peptides, in particular muramyl peptides, ligands of innate immunity, which can be used to correct pathological conditions in humans [8,9,10,11,12,13,14,15].

**Figure 1:**
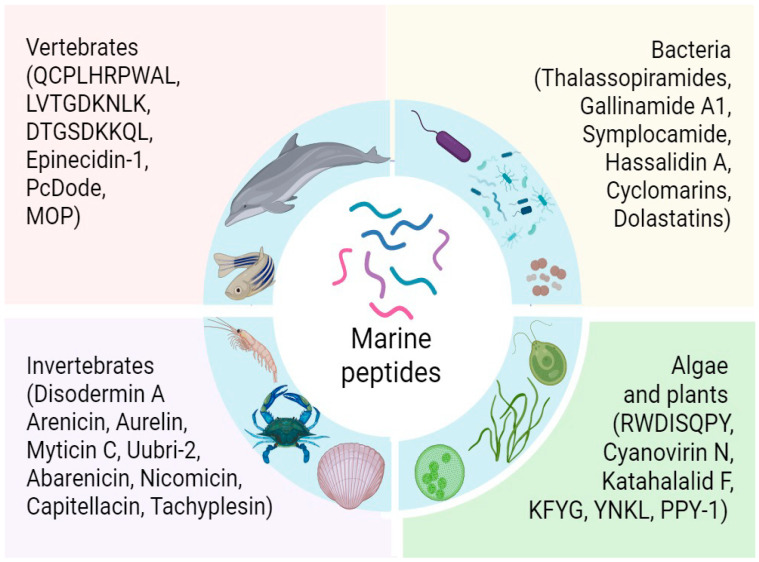
Sources of marine peptides.

Marine peptides produced by various marine organisms such as marine bacteria, algae, plants, sponges, invertebrate and vertebrate animals offer promising solutions to numerous health challenges due to their unique structures and mechanisms of action [16] (Figure 1).

Discovery and study of marine peptides have evolved significantly over the past few decades [17]. Initially recognized for their antimicrobial properties, marine peptides have since been found to possess a wide range of bioactivities [18,19,20]. Marine peptides exhibit various therapeutic activities due to their ability to interact with biological membranes, modulate immune responses, inhibit or activate specific enzymes and receptors, etc. [21,22,23]. Samples of marine organisms for obtaining antimicrobial peptides are collected from diverse environments, including deep sea regions and coastal areas [23,24,25]. Peptides are isolated through a combination of extraction, isolation, and purification methods. Bioengineering methods are also used for maintaining wild-type peptide modified analogs [26]. Structural characterization of peptides involves amino acid sequencing techniques, mass spectrometry, nuclear magnetic resonance (NMR) spectroscopy. Bacterial expression systems for production of recombinant peptides and their stable isotope-labeled analogue have been developed to determine their three-dimensional structures and investigate the mechanisms of action [27]. Different bioactivity assays have been used, including antibacterial, antifungal, antiviral, and cytotoxicity tests, among others, to evaluate the therapeutic potential of isolated peptides. The development of omics technologies has increased significantly the number of new marine peptides discovered in silico with confirmation of their biological activity in the experiment [28,29].

Marine peptides can be synthesized by ribosomal and non-ribosomal synthesis. The animal marine peptides are directly synthesized in ribosomes as distinct from bacterial peptides which are products of secondary metabolism. A great majority of the animal marine peptides are synthesized as preproteins which are further processed with the formation of active molecules. Prodomains of the precursor proteins are located between signal and mature peptide sequences or in *C*-terminal regions and, as a rule, bear negative charges. For example, the nicomicin precursor includes the BRICHOS domain within an acidic pro-region [30]. Marine peptides can also be represented by derivatives of N-terminal or C-terminal regions of proteins, in particular histones [31,32].

Marine peptides are being actively studied due to their potential in the treatment of infectious and non-infectious diseases, in particular as an alternative to antibiotics or as agents administered together with them [18,33]. In addition, they can be used in agriculture to protect plants and animals from pathogens, or in other economy sectors, for example, with a view to protect bioreactors from microbial fouling [34,35].

To date, several hundreds of marine peptides have been identified, each exhibiting unique structure and function. Their multivarious activities and therapeutic potential are being exemplified and discussed in this review.

## 2. Antimicrobial Activity

### 2.1. Antibacterial

Marine peptides have shown a strong antibacterial activity against a variety of pathogenic bacteria, including multi-drug resistant strains. These peptides often function by disrupting bacterial membranes, leading to cell lysis and death. Some of them have LPS-binding properties and reduce LPS-induced inflammation [36].

Antimicrobial peptides may be unique, occurring in a certain type of marine organism, or may be present in different species. For example, arenicin isolated from the lugworm *Arenicola marina* is unique to this species. The peptide has demonstrated potent activity against both Gram-positive and Gram-negative bacteria [37,38,39,40,41]. At the same time, antimicrobial peptide hepcidins are found not only in many marine vertebrates, especially fish, but also in land animals, including humans, and they not only protect against infection but also participate in iron transfer [42].

Hepcidin, isolated from Japanese sea bass *Lateolabrax japonicus*, has a broad spectrum of antimicrobial activity against Gram-negative and Gram-positive bacteria [43]. Hepcidin employs multiple molecular mechanisms to eliminate microbes. Like many other cationic AMPs, hepcidin binds to negatively charged bacterial cell membranes via electrostatic interaction. This can disrupt membrane integrity by forming transient pores or carpet-like lesions, leading to leakage of cellular contents and, as a result, to bacterial cell death [44]. Electron microscopy observations confirm that hepcidin causes morphological damage and even aggregation of bacterial cells, consistent with membrane-targeted killing. Hepcidin may also translocate into microbes: a conserved metal-binding motif (ATCUN) at its N-terminus enables hepcidin to complex with Cu(II) ions, generating reactive oxygen species that damage bacterial DNA and proteins [45]. Furthermore, disulfide-stabilized β-sheet structure of the peptide is crucial to its function. The intact hepcidin can bind microbial DNA, whereas reduced or cysteine-alkylated forms lose DNA-binding abilities and bactericidal activities. In vertebrate hosts, hepcidin plays an additional role of hormonal iron regulation: it binds to the iron exporter ferroportin on the cell surface, causing its internalization and degradation [46]. This traps iron inside cells and deprives extracellular bacteria of iron, an essential growth nutrient [47]. Thus, hepcidin defends against infection through direct microbial killing and by starving pathogens of iron. The latter action specific for hepcidin.

Antimicrobial peptides from marine Polychaeta—capitellacin from *Capitella teleta*, abarenicin from *Abarenicola pacifica*, UuBRI-21 from *Urechis unicinctus*, nicomicin from *Heteromastus filiformis*—demonstrate a wide range of antibacterial activities including antibiofilm one [30,48,49,50]. Plicatamide, the antimicrobial octapeptide from the solitary tunicate *Styela plicata* hemocytes, induces increase in potassium efflux, cessation of oxygen consumption, and loss of viability within seconds in methicillin-resistant and wide-type of *S. aureus* [51]. Halocycin from hemocytes of the tunicate *Halocynthia aurantium* is active not only against methicillin-resistant *Staphylococcus aureus*, but also against multiresistant *Pseudomonas aeruginosa* [52].

Some antimicrobial peptides are present in organisms at a certain stage of their development. For example, botryllin, isolated from the morulae of the colonial ascidian *Botryllus schlosseri*, has a high activity against *S. epidermidis* and *S. cerevisiae*. The peptide is synthesized by circulating cytotoxic morula cells, stored inside their granules and released upon morula cell degranulation [53].

### 2.2. Antibiofilm

The ability of marine peptides to inhibit biofilm formation is significant, as biofilms contribute to bacterial resistance and chronic infections. Biofilm inhibition can occur through multiple mechanisms, such as preventing the initial adhesion of bacteria to surfaces or disrupting established biofilms. For instance, arenicin from *Arenicola marina* prevents the formation of biofilms by *Pseudomonas aeruginosa* [54].

Another example is lipopeptide from the marine sponge-derived bacterium *Bacillus licheniformis* which has shown efficacy in preventing biofilm formation by different bacteria including human pathogens *Staphylococcus aureus* and *Escherichia coli* [55]. *Bacillus licheniformis* has been shown to inhibit the fouling of marine organisms, particularly *Bugula neritina*, under natural conditions [56]. The use of compounds that prevent biofilm formation is possible not only in medicine, but also in biotechnology and pharmaceutical industry to protect bioreactors from fouling [57,58].

### 2.3. Antifungal

Antifungal peptides from marine sources target fungal cell membranes or interfere with intracellular processes, leading to cell death. Examples include discodermin from the sponge *Discodermia kiiensis* and tachyplesin from hemocytes of the horseshoe crab *Tachypleus tridentatus*, which have been shown to exhibit antifungal activity against *Candida albicans* [59,60,61,62].

Dolastatins are peptides originally isolated from the Indian Ocean bearded seal *Dollabella auricularia*. They have been shown to display a high antifungal activity against *Cryptococcus neoformans* and antiproliferative properties consisting in inhibition of tubulin polymerization [63]. As it has turned out later, dolastatins are synthesized by cyanobacteria presenting in the food of the bearded seal and have not only antibacterial but also antitumor activities [64,65]. Kahalalide F, depsipeptide isolated from the Hawaiian sea slug *Elysia rufescens* and from the green alga *Bryopsis pennata*, is a potent antifungal agent against *C. albicans*, *C. neoformans*, and *Aspergillus fumigatus* [66]. Epinecidin-1 from the orange-spotted grouper *Epinephelus coioides* suppresses the phytopathogenic fungus *Botrytis cinerea* and gray mold on peach fruits after harvest [31,32]. Lipopeptide Hassallidin A from the cyanobacterium *Hassallia* sp. exhibits an antifungal activity against *A. fumigatus* and *C. albicans* [67,68].

### 2.4. Antiviral

Marine organisms live in constant contact with pathogens, including viruses, and have a reliable protection against them in the form of ribonucleases and antiviral peptides [69,70]. A large number of marine peptides with an antiviral activity have been identified. In particular, the peptide that protects shrimp from the white spot syndrome virus (WSSV) has been identified in the Pacific white shrimp *Litopenaeus vannamei* [71].

Myticin C protects the midi *Mytilus galloprovincialis* from the ostreid herpes virus and from the fish rhabdovirus and has a pronounced activity against the human herpes viruses HSV-1 and HSV-2 [72,73,74]. Interestingly, myticin C has more than a hundred variants of nucleotide sequences in different species of mollusks, which indicates an important role of the peptide in protecting mussels [75].

Marine peptides have shown antiviral properties by interacting with the viral capsid, preventing virus penetration into host cells or viral replication. For example, the peptide cyanovirin-N, isolated from the blue-green alga *Nostoc ellipsosporum*, has been shown to inhibit HIV by binding to glycoproteins of the viral envelope [76]. Another mode of antiviral action is inhibition of the NF-κB-dependent HIV-1 replication by bengamide A, first isolated from the sponge *Jaspis* cf. *coriacea* [77,78,79]. Depsipeptides from the marine sponge *Stelletta* sp. and the peptide inhibitor of the HIV-1 integrase from marine polychaetes also exhibit anti-HIV activity [80,81].

Marine peptides from the tuna *Thunnus obesus* and the brown algae *Undaria pinnatifida* have shown antiviral activities against the SARS-CoV-2 virus [82,83]. These peptides bind to hACE2 and interrupt the binding of SARS-CoV-2 spike proteins to ACE [82]. The cyclic depsipeptide dehydrodidemnin B, originally isolated from the tunicate *Aplidium albicans*, has also been reported to have a neutralizing activity against SARS-CoV-2 at nanomolar concentrations and is significantly more potent than remdesivir [84]. A large number of cyclic peptides from the tunicate *Trididemnum solidum*, the marine sponge *Theonella swinhoei*, and the marine cyanobacteria *Schizothrix* (Gallinamide A 1) have been identified and shown to have SARS-CoV-2 inhibitory properties [85,86,87].

### 2.5. Antiparasitic

An antiparasitic activity of marine peptides is manifested by their effectiveness against protozoan parasites and helminths [88]. The cyclic peptide jasplakinolide, also named jaspamide, has been isolated from the sponge species *Jaspis* and shown to inhibit the *P. falciparum* growth and impair host cell invasion [89,90]. The mechanism of jasplakinolide action involves the reduction in actin subunits which are necessary to drive polymerization and stabilization of parasite actin filaments [91,92]. Jasplakinolide at micromolar concentrations also reduced the motility and invasiveness of *Toxoplasma gondii* and suppressed the growth and encystation of *Entamoeba histolytica* and *Entamoeba invadens*, causing the formation of F-actin aggregates [93,94,95].

Cyclic peptides cyclomarins from extracts of the marine bacterium *Streptomyces* sp. exhibited an antiparasitic activity against multidrug-resistant *P. falciparum* strains in the nanomolar concentration range [96,97]. Their mechanism of action is based on binding the parasite’s diadenosine triphosphate hydrolase, without affecting the human homologue [96]. The peptide symplocamide from the marine cyanobacterium *Symploca* sp. also inhibited *P. falciparum* to a significant extent and, to a lesser extent, *T. cruzi* and *L. donovani* [98].

Marine peptides have demonstrated anthelmintic properties. For example, the peptide fraction from the marine sponge *Hymeniacidon heliophila* has demonstrated an anthelmintic activity against the parasitic worm *Haemonchus contortus*, which is an important cause of disease in livestock [99].

A total of 25 marine-derived cyclic peptides with antiparasitic activity were found by a systematic analysis of the scientific publications. Antimalarial activity is the most reported (51%), followed by antileishmanial (27%) and antitrypanosomal (20%) activities [88].

## 3. Immunomodulatory Activities

Modulation of immune responses by marine peptides can enhance the human body’s defense mechanisms via modulating action on innate and adaptive immunity and maintaining homeostasis. The study of an immunomodulatory activity of marine peptides is of particular importance in connection with determining possibilities of their use in medicine. Marine peptides can affect both cellular and humoral immunity, modulating a functional activity, interaction of immune cells and production of cytokines, chemokines, and growth factors by them.

In particular, thalassospiramides A and D from the marine bacteria *Thalassospira* sp. suppress LPS-induced NO production, inhibit IL-5 expression in TH-2-mediated inflammatory diseases. These peptides might be used as drugs in TH-2-mediated diseases, such as asthma [100,101,102].

The peptide PPY1 from the marine algae *Pyropia yezoensis* completely inhibited the LPS-stimulated NO release and reduced the release of pro-inflammatory cytokines (inducible NO synthase, cyclooxygenase-2, IL-1β and TNF-α in LPS-stimulated macrophages) [99]. PPY1 was suggested as a potential drug with the significant anti-inflammatory activity [103,104].

The pentadecapeptide RVAPEEHPVEGRYLV from the clam *Cyclina sinensis* (SCSP) in a cyclophosphamide (CTX)-induced murine model of immunosuppression significantly increased the production of serum IL-6, IL-1β, and tumor necrosis factor TNF-α [105]. Moreover, SCSP treatment enhanced the proliferation of splenic lymphocytes and peritoneal macrophages, as well as phagocytosis of the latter in a dose-dependent manner. SCSP also elevated the phosphorylation levels of p38, ERK, JNK, PI3K, and Akt, and up-regulated IKKα, IKKβ, p50 NF-κB, and p65 NF-κB protein levels while down-regulating IκBα protein levels [105].

The peptide EP from the European eel *Anguilla anguilla* activated macrophages to produce NO and iNOS and promoted TNF-α and IL-6 secretion in a concentration-dependent manner. Moreover, EP dose-dependently activated NF-κB and MAPK signaling pathways [106].

### 3.1. Anti-Inflammatory

Peptides exhibiting anti-inflammatory effects reduce inflammation markers in cell models. This can be beneficial in treating chronic inflammatory conditions.

A large number of peptides have been obtained from hydrolysates of marine organisms. Various databases are used to select the most promising ones, which can predict biological activity based on their structural features. The database http://i.uestc.edu.cn/AntiDMPpred/cgi-bin/AntiDMPpred.pl (accessed on 12 April 2025) can predict, for example, anti-inflammatory or antidiabetic activity based on the peptide structure [107,108]. This approach allowed to identify the peptide QCPLHRPWAL from mesopelagic fish, including *Maurolicus muelleri* and *Benthosema glaciale*, which had anti-inflammatory and analgesic activities [107]. The peptide KC14 from the common carp *Cyprinus carpio* demonstrates anti-inflammatory and anti-oxidant activities by scavenging free radicals in CuSO_4_-exposed groups, highlighting its potential as a protective agent against metal-induced oxidative stress and inflammation, thus offering insights into its therapeutic application in environmental toxicology [108].

### 3.2. Modulation the Complement System

Arenicin-1 and its derivative Ar-1-(C/A) from the marine worm *Arenicola marina* exhibited interesting properties; at low concentrations it activated the complement system, while at high concentrations the peptide inhibited it [109,110,111,112,113].

### 3.3. Chemotactic

Marine peptides exhibit chemotactic activity, attracting immune cells to sites of infection or injury. This property is crucial for initiating an effective immune response. For example, fish antimicrobial peptides piscidins exhibit not only antimicrobial but also immunomodulatory activity. Four piscidins from the orange-spotted grouper *Epinephelus coicodes* (ecPis1S, ecPis2S, ecPis3S, and ecPis4S) exhibited chemotactic activity toward head kidney leukocytes [114]. The most potent chemotactic activity was observed for ecPis2S. In addition, *E. coicodes* piscidins enhanced the respiratory burst and phagocytic activity of macrophages, as well as mRNA expression levels of chemokine receptors, Toll-like receptors, T-cell receptors, and proinflammatory cytokines [114]. Piscidins were found in the gills and skin of fish and were responsible for neuroimmune interactions at barrier surfaces [115].

### 3.4. Opsonizing

Enhanced phagocytosis of pathogens by immune cells was noted for marine peptides with an opsonizing activity. These peptides can bind to the surface of pathogens, marking them for destruction by phagocytes. An example includes marine-derived antimicrobial peptides that can enhance the opsonization of bacteria, leading to more efficient clearance them by immune cells. Crustins have been found in the hemolymph of the subphylum Crustacea members, including crabs, shrimps, and lobsters [115,116,117,118,119]. Crustins enhance phagocytosis by opsonizing bacteria, leading to more effective anti-infective defenses [120,121,122]. Knockdown of the crustin II gene increases mortality in animals infected with the bacterial pathogen, but not with the fungal one [123].

## 4. Anticancer Activity

Marine peptides are being actively studied as potential anticancer drugs. When studying the mechanism of their anticancer action, it was found that they can realize different strategies to destroy tumor cells [124,125,126,127].

Marine peptides induce apoptosis in cancer cells and inhibit tumor growth in vitro [128]. They can trigger programmed cell death through mechanisms such as mitochondrial disruption, caspase activation, and inhibition of angiogenesis [126,129]. For instance, the peptides hemiasterlins from *Auletta* sp. and *Siphonochalina* spp. sponges have demonstrated a potent anticancer activity by inhibiting cell division via mitotic arrest and abnormal spindle formation [130].

The cyclic peptide symplocamide from the marine cyanobacterium of *Symploca* sp. exerted a cytotoxic activity against H-460 lung cancer cells and neuroblastoma neuro-2a cells via inhibition of serine proteases [98].

Selective cytotoxicity against cancer cell lines without affecting normal cells is a notable property of some marine peptides. The peptide dolastatin 10 from the sea hare *Dolabella auricularia* has shown a significant cytotoxicity against various cancer cell lines and has been used as a template for developing anticancer drugs [64,65].

Tachyplesins, isolated from the horseshoe crab *Tachypleus tridentatus*, in addition to their antimicrobial activity, exerts a cytotoxic activity toward human cancer cells, including non-small-cell lung cancer cells, by inducing apoptosis [129,130,131,132,133,134]. Tachyplesins enhance the chemosensitivity of cancer cells to cisplatin, reducing the effective concentrations of cisplatin [132,133,134]. Polyphemusin III from the horseshoe crab *Limulus polyphemus* disrupts the plasma membrane integrity and induces cell death that is apparently not related to apoptosis [135].

Didemnins are depsipeptides isolated from the Caribbean tunicate *Trididemnum solidum* [136,137]. Didemnins realize their antitumor activities against human cancer cells via protein synthesis inhibition [138,139,140,141].

Piscidin-1 derived from the mast cells of hybrid striped bass (*Morone saxatilis* × *M. chrysops*) inhibits angiogenesis and induces apoptosis in oral squamous cell carcinoma through reactive oxygen species production [142].

Antioxidant effect of marine oligopeptide preparation (MOP) from the chum salmon *Oncorhynchus keta* determined in radiation injured mice MOP has been suggested as a potential drug to alleviate radiation-induced oxidation damage in cancer patients [143].

Summarized data on activities of selected marine peptides are presented in Table 1.

## 5. Other Activities

### 5.1. Antihypertensive

Blood pressure-lowering effects through inhibition of angiotensin-converting enzyme (ACE) have been observed in marine peptides. For example, peptides derived from fish proteins have demonstrated ACE inhibitory activity, making them potential candidates for managing hypertension [141,142,143,146,147,148,149]. In particular, the dipeptide Val-Tyr, derived from sardine muscle hydrolysate, and oligopeptides isolated from kaciobushi (simmered, smoked, and fermented skipjack tuna) have demonstrated antihypertensive effect and recommended as a food supplement to maintain blood pressure [147,148,149].

Two peptides Leu-Val-Thr-Gly-Asp-Asp-Lys-Thr-Asn-Leu-Lys (LVTGDKTNLK) and Asp-Thr-Gly-Ser-Asp-Lys-Lys-Gln-Leu (DTGSDKKQL) from the muscle of the small red scorpionfish *Scorpaena notata* have shown high antioxidative and angiotensin I converting enzyme inhibitory activities [150]. Dipeptides Val-Tyr, GND Ile-Tyr, Phe-Tyr, and Ile-Trp from the brown algae *Undaria pinnatifida* have shown a significant antihypertensive effect after oral administration to spontaneously hypertensive rats [151]. Peptides Ala-Ile-Tyr-Lys (AIYK), Tyr-Lys-Tyr-Tyr (YKYY), Lys-Phe-Tyr-Gly (KFYG), and Tyr-Asn-Lys-Leu (YNKL) from the brown algae *Undaria pinnatifida* exerted antihypertensive effects via inhibition of ACE when administered orally [144].

Peptide Arg-Trp-Asp-Ile-Ser-Gln-Pro-Tyr from the brown algae *Sargassum maclurei* has shown endothelin-1 suppressing and antihypertensive activities [145].

Antihypertensive peptides inhibiting ACE were isolated from the ribbonfish *Trichiurus lepturus*. In silico screening yielded five peptides: FAGDDAPR, QGPIGPR, IFPRNPP, AGFAGDDAPR, and GPTGPAGPR. Three of them—FAGDDAPRR, QGPIGPR, and GPTGPAGP—showed good ACE-inhibitory effects, thus underscoring their therapeutic potential [152].

### 5.2. Antinociceptive

Cone snails are a source of several hundred peptides (conopeptides), among which peptides for the treatment of severe pain have been identified [153,154]. Venom peptides target a wide variety of membrane-bound ion channels and receptors and are important molecular tools for the study of Ca^2+^-, K^+^-, Na^+^-, Cl—channels, and a large number of receptors (nicotinic ACh receptor α7, natriuretic peptide receptor A, α_1B_-adrenoceptor, noradrenaline transporter and others) [155,156]. The conopeptide MVIIA or Prialt has been clinically tested and is used as the drug to treat severe pain [18,157,158].

### 5.3. Wound Healing

Acceleration of wound healing and tissue regeneration is a critical therapeutic application of marine peptides. Marine peptides have shown significant wound-healing properties by promoting angiogenesis, cell migration, and proliferation. In particular, peptides from the skin of the Nile tilapia *Oreochromis niloticus* accelerated wound healing in laboratory animals [159]. There was an increase in the production of nitric oxide (NO), hydrogen peroxide (H_2_O_2_), proinflammatory (IL-2 and IFNγ), and anti-inflammatory (IL-4) cytokines, as well as cell proliferation markers PCNA and KI67. In addition, there was an increase in the formation of new vessels [159]. Interesting studies have been conducted on the effect of collagen peptides from fish skin on the wound-healing process in pregnant rats undergoing cesarean section [160]. Oral administration of peptides after cesarean section increased skin tensile strength, uterine rupture pressure, hydroxyproline content, CD34, and connective tissue growth factor expression, as well as collagen and smooth muscle fiber formation [160].

It is noted that fish skin is a source of biocompatible and biodegradable compounds and does not require frequent dressing changes. It also displays wound-healing acceleration, antibacterial, and antiviral properties, and is not associated with disease transmission or adverse immunological reactions [161].

In addition, several peptides derived from marine invertebrates have also been found to have wound-healing properties. For example, myticin C from the marine mussel *Mytilus galloprovinciali* promoted wound healing in laboratory animals through angiogenesis and re-epithelialization [162]. Halocydin from the tunicate *Halocynthia aurantium* promoted tissue proliferation, angiogenesis, and regeneration in a mouse model of methicillin-resistant *S. aureus*-infected surgical wound when applied topically while also exerting antimicrobial activity [163].

### 5.4. Antiaging

The task of healthy longevity is becoming relevant with the increase in human life expectancy. To date, a large number of marine peptides have been discovered. In animals, they have shown the ability to slow down the aging process along with resveratrol, metformin, rapamycin, and aspirin [164,165]. Their antioxidant activity and the ability to restore immune, hormonal, and mitochondrial functions and correct the microbiome have been noted [164]. Antioxidant properties and enhancement of cell repair mechanisms are crucial for the antiaging effect. Marine peptides can remove free radicals, reducing oxidative stress and promoting cell repair. Peptides from the sea cucumbers *Cucumaria rondose* have shown a significant antioxidant activity in aging fruit flies and mice with neurodegenerative diseases [165,166,167,168]. The sea cucumber peptides effectively extended the lifespan of fruit flies and improved D-galactose-induced learning impairment in aging mice by inhibiting lipid peroxidation, upregulating the expression of the aging suppressor gene Klotho, and by enhancing the activities of superoxide dismutase and glutathione peroxidase [168,169]. Peptides from the sea cucumber *Apostichopus japonicus* enhanced the survival of nematodes under oxidative stress and reduced the levels of reactive oxygen species (ROS), increased the activities of superoxide dismutase and catalase, reduced aging pigments level, and increased lifespan [166,167].

Laxaphycins from the cyanobacterium *Anabaena torulosa* affect mitochondrial function through AMPK phosphorylation and mTOR inhibition [170]. Peptides from the sea cucumber *Acaudina leucoprocta* significantly modulate relative levels of muscle glycogen oxidative stress protein, energy metabolism, and mitochondrial function by regulating NRF2 and AMPK signaling pathways, significantly improving exercise performance and exerting anti-fatigue effects in laboratory animals [171].

### 5.5. Anti-Photoaging and Skin Protection

Anti-photoaging marine peptides inhibit oxidative stress damage. Peptides ICRD and LCGEC, derived from tuna eggs, exhibit DPPH free-radical scavenging activity and protect HaCaT cells from ultraviolet B (UVB) radiation [172]. The abalone peptide ATPGEG mitigates UVB-induced ROS levels in HaCaT cells and inhibit DNA damage by UVB exposure [173]. Three antioxidant peptides PKK, YEGGD, and GPGLM from the bonipjack heart artery balls of Skipjack tuna effectively eliminate ROS and reduce intracellular malondialdehyde (MDA) levels [174]. These findings demonstrate that marine peptides can effectively neutralize free radicals, thereby preventing UV-induced damage. This is of great value for potential application of marine peptides in cosmetology and primary skin disease prevention.

### 5.6. Antiplatelet and Antithrombotic

Cyclodepsipeptide isaridin E from the marine-derived fungus *Amphichorda felina* (syn. *Beauveria felina*) inhibits ADP-induced platelet aggregation, activation, and secretion via the PI3K/Akt signaling pathway. In addition, it reduces thrombosis formation in the FeCl_3_-induced mouse carotid model without increasing the bleeding time. These finding suggest that isaridin E has potent antithrombotic properties without increasing the risk of bleeding [175].

## 6. Discussion

Marine organisms are an inexhaustible source of biologically active compounds, including peptides. Marine bacteria, algae, plants, and animals contain peptides that can be used by humans as medicines and biologically active supplements. The possibility of expressing their recombinant analogs and/or introducing modifications into ribosomally synthesized peptides significantly expands the possibilities of obtaining peptides with specified properties.

**Figure 2:**
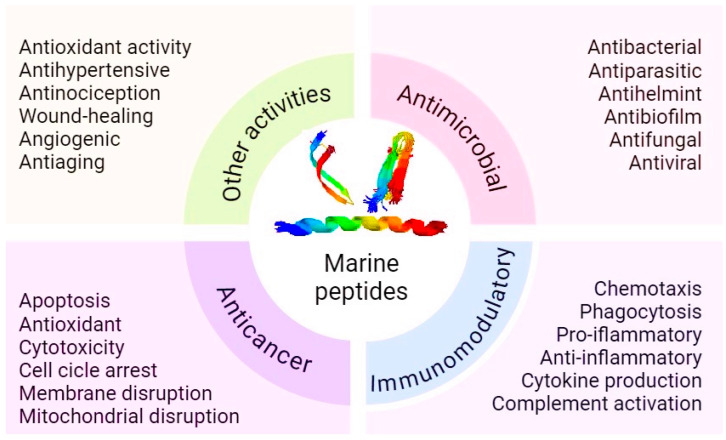
Biological activities of marine peptides.

The diverse bioactivities of marine peptides highlight their potential as versatile therapeutic agents (Figure 2). Molecular mechanisms behind these activities, such as membrane disruption, enzyme inhibition, receptor modulation, etc., are explored. For example, antibacterial activities of marine peptides often involve disruption of bacterial cell membranes, leading to cell lysis. Their antiviral properties may result from binding to viral envelope proteins, preventing virus entry into host cells. Particular attention is paid to marine antimicrobial peptides as potential means of combating antibiotic-resistant strains of bacteria, as well as candidate medicines against viral and parasitic diseases.

Antimicrobial peptides (AMPs) are ancient molecular factors of innate immune system which provide the first-line defense of the host against pathogen penetration. The first bacteria that emerged in the ocean more than 4 billion years ago used AMPs, and this defense still ensures their viability and prosperity [176,177,178]. Many studies have shown that marine peptides can exhibit more potent bioactivities compared to their terrestrial counterparts due to their unique structures adapted to the marine environment.

By comparing aquatic and terrestrial AMPs both of which are integral to host defenses across different ecosystems, it is crucial to note that they exhibit some distinct strengths and limitations. Both aquatic and terrestrial AMPs typically have broad-spectrum antimicrobial activities against bacteria, fungi, and some viruses. Marine AMPs, however, often show exceptional breadth and potency, likely reflecting the immense microbial diversity of oceans. The marine environment’s pressures induce production of AMPs with activities against a wide range of pathogens. Terrestrial AMPs, for example human defensins and insect cecropins, are also broad-spectrum peptides, but some of them are more specialized to organisms’ typical pathogens. Overall, neither category is universally superior in efficacy, but marine AMPs provide a novel arsenal with potentially broader or unique targets. For instance, some marine peptides interfere with microbial biofilms or virulence mechanisms which are not addressed by terrestrial peptides. This means that marine AMPs may retain activities against drug-resistant strains with minimal cross-resistance.

A notable advantage of marine AMPs is their adaptation to harsh conditions. Evolved in high-salinity, extreme environments, many marine peptides remain active both in physiological and in elevated salt conditions. For example, fish-derived AMPs pleurocidin and epinecidin retain antibacterial activity at salt concentrations that inactivate mammalian AMPs [179,180]. Terrestrial AMPs often lose potency in high-salt solutions or protease-rich settings. The human beta-defensin-1, for instance, is rendered ineffective by the salt levels in airway surface fluid. Marine peptides commonly feature chemical modifications; for example, the presence of D-amino acids, bromination or cyclization enhance their proteolytic stability. Some terrestrial AMPs are disulfide-stabilized (defensins) or cyclic (plant cyclotides), but they are frequently linear and unmodified, hence more susceptible to degradation. On the other hand, those evolutionary optimizations make marine AMPs structurally more complex, which can complicate their synthesis and formulation.

Terrestrial and marine AMPs often share common mechanisms of action (membrane disruption, pore formation, immunomodulation, etc.), but many marine AMPs possess novel structural motifs that can translate into unique modes of action. Terrestrial AMPs have co-evolved with microbes in land- or host-associated environments; some pathogens have developed partial resistance or regulatory mechanisms to avoid certain vertebrate AMPs. In contrast, marine AMPs have originated from organisms such as marine invertebrates, fish, etc., that interact with multivarious microorganisms. This can yield mechanisms of action to which human pathogens have never been exposed, resulting in a lower tendency to induce or encounter antimicrobial resistance. Both marine and terrestrial AMPs generally have a relatively limited risk of resistance development because they often target fundamental microbial membrane structures. However, bacteria in terrestrial settings may secrete proteases or binding proteins to neutralize host AMPs; such resistance mechanisms might be less effective against entirely foreign marine AMPs. In practice, each AMP must be evaluated individually, but the evolutionary success of marine AMPs is a clear asset in the fight against multi-drug-resistant microbes.

Despite the numerous advantages of marine AMPs over AMPs from terrestrial organisms, marine AMPs have a number of limitations [181]. One of these issues is relatively low stability and bioavailability. Peptides are generally prone to degradation by proteases and may have poor pharmacokinetics. In the body, digestive enzymes and blood proteases can quickly break down peptides, and a majority of them cannot survive in the acidic stomach environment if taken orally. Marine peptides are no exception, despite some having robust structures, like cyclic or D-amino acid-containing peptides that resist proteolytic cleavage. Thus, stability of marine AMPs remains a major concern [182]. They can be degraded during processing, storage, or after administration, which may reduce their activities [183]. Additionally, large peptides often face problems when cross cell membranes or the gut lining, thus limiting bioavailability. To address this, researchers have developed formulation strategies such as encapsulation, liposomes, or peptide stapling to protect them and enhance their delivery [184].

A critical perspective of any drug is its safety and toxicity. Many AMPs are touted as having a low toxicity toward human cells and preferentially targeting microbial membranes. Generally, it is the case that marine peptides often have a low (or no) toxicity at therapeutic doses, and they tend to be less allergenic than some small-molecule drugs [185,186]. Nonetheless, some of them can damage host tissues or cause side effects. In vitro tests have shown that certain AMPs are hemolytic at high concentrations, and some marine peptides can trigger histamine release or cytokine storms if not carefully controlled. Therefore, toxicity and immunogenicity must be thoroughly assessed for each peptide. Even if marine peptides are “less allergenic”, one should confirm that they do not provoke immune reactions before using them in foods or medicines. To overcome this challenge, marine peptides can be engineered or modified to obtain peptides with better specificity: medicinal chemists can alter certain residues to reduce toxicity while retaining antimicrobial action [187,188]. This kind of structure–activity relationship (SAR) study is ongoing for numerous peptides, including shortening peptides to the minimum active fragment and thereby reducing off-target effects.

The use of systems biology approaches and existing databases of peptides from marine organisms allows us to identify new biologically active compounds and determine the possibilities of their use [189,190,191,192]. With the use of big data-based and multi-omics technologies, great progress can be achieved in the search of novel marine peptides which may be developed as promising pharmaceuticals.

Marine peptides are increasingly incorporated into cosmeceuticals for skin health, owing to their safety and bioactivity. One high-potential application of marine AMPs is anti-aging skincare. Marine collagen peptides, typically derived from fish skin or scales, are used as oral supplements and topical additives to improve skin elasticity and reduce wrinkles. These collagen-derived peptides supply essential amino acids such as glycine, proline, and others that support the dermal extracellular matrix. Recent studies have shown that ingesting marine collagen could increase dermal collagen content, improve skin laxity, and enhance elasticity, leading to visibly smoother, hydrated skin [193]. Such supplements have proven relatively effective action in mitigating signs of skin aging [194]. In addition, marine collagen materials have shown a utility in wound healing by acting as biomimetic scaffolds that promote cell attachment and tissue regeneration, further supporting their therapeutic value in dermatology [195].

Marine algae also produce peptides with antioxidant and anti-inflammatory properties. For instance, brown algae extracts contain peptides that protect skin against UV-induced oxidative stress and collagen degradation [196]. These peptides are incorporated into anti-aging creams and sunscreens in order to prevent photoaging and inflammation in the skin. Marine peptides hold promise for developing new drugs and therapies, particularly in areas where conventional treatments are ineffective [197,198,199].

Their broad spectrum of activities makes them suitable candidates for treating multidrug-resistant infections, cancer, inflammatory diseases, and more. The use of AMPs as part of combination therapy will allow to amend conventional antimicrobial drugs, improve their clinical outcomes, reduce toxicity and, most importantly, prevent the development of resistance [200,201,202,203].

In addition, marine peptides can be used not only in medicine but also for the control over microbial expansion of industrial importance, in particular in aquaculture [28,104,204].

## 7. Conclusions

Marine peptides exhibit a wide range of biological activities with significant therapeutic potential. Their unique properties and diverse mechanisms of action make them promising candidates for developing new medicines. Continued research and development are essential to harness their full potential. Future studies should focus on large-scale synthesis, recombinant production, detailed mechanism studies, and clinical evaluations and translation of novel findings into practical applications. Investigation of the potential synergistic effects of combining marine peptides with existing therapies to enhance their efficacy is of the importance as well. Sustainable sourcing of marine peptides and harvesting marine organisms must be in the zone of special attention. This valuable biological resource provides a strong foundation for in-depth therapeutic applications of marine peptides and drug development on their basis.

## Figures and Tables

**Table 1:** Selected examples of marine peptides and their multivarious activities.

Taxa	Source Organism	Peptide	Activity	References
Microorganisms	Marine bacteria *Thalassospira* sp.	Thalassospiramides A and D	Immunomodulation: suppress LPS-induced NO production, inhibit IL-5 expression in TH-2-mediated inflammatory diseases	[100,101,102]
	Cyanobacterium *Schizothrix*	Gallinamide A 1	Immunomodulation: inhibits SARS-CoV-2	[87]
	Cyanobacteria *Symploca* sp.	Symplocamide	Antiparasitic activity: inhibits *P. falciparum*, *T. cruzi* and *L. donovani*	[98]
	Sponge-derived bacterium *Bacillus licheniformis*	Lipopeptide	Antibiofilm activity against *Staphylococcus aureus*, *Escherichia coli*, and *Bugula neritina*	[55]
	Bacterium *Streptomyces* sp.	Cyclomarins	Antiparasitic activity against multidrug-resistant *P. falciparum*	[96,97]
	Bearded seal-derived cyanobacteria	Dolastatins	Antifungal activity against *Cryptococcus neoformans*; antiproliferative activity (inhibition of tubulin polymerization); antitumor activity	[63,64,65]
	Cyanobacteria *Hassallia* sp.	Hassallidin A	Antifungal activity against *A. fumigatus* and *C. albicans*	[67,68]
Algae and plants	Brown algae *Undaria pinnatifida*	AIYK, YKYY, KFYG, and YNKL	Antihypertensive activity (inhibition of ACE)	[144]
	Brown algae *Sargassum maclurei*	RWDISQPY	Antihypertensive activity; endothelin-1 suppressing activity	[145]
	Sea slug *Elysia rufescens*, green algae *Bryopsis pennata*	Kahalalide F	Antifungal activity against *C. albicans*, *C. neoformans*, and *Aspergillus fumigatus*	[66]
	Blue-green alga *Nostoc ellipsosporum*	Cyanovirin-N	Antiviral activity: inhibits HIV by binding to glycoproteins of the viral envelope	[76]
	Marine algae *Pyropia yezoensis*	PPY1	Anti-inflammatory acivity: inhibits LPS-stimulated NO release, reduces the release of proiflammatory cytokines in LPS-stimulated macrophages	[99,103,104]
Invertebrates	Mussel *Mytilus galloprovincialis*	Myticin C	Antiviral activity against herpesviruses	[72,75]
	Jellyfish *Aurelia aurita*	Aurelin	Antimicrobial activity	[21,27]
	Polychaeta Nicomache minor	Nicomicin	Antimicrobial activity	[30]
	Polychaeta *Capitella teleta*	Capitellacin	Antimicrobial and antibiofilm activities	[48,49,50]
	Polychaeta *Arenicola marina*	Arenicin	Antimicrobial activity; influence on complement system	[37,38,39,40,41,54,110,111,112,113]
	Polychaeta *Abarenicola pacifica*	Abarenicin	Antimicrobial and antibiofilm activities	[48,49,50]
	Polychaeta *Urechis unicinctus*	UuBRI-2	Antimicrobial and antibiofilm activities	[48,49,50]
	Porifera sponge *Discodermia kiiensis*	Discodermin A	Antifungal activity against *Candida albicans*	[59,60]
	Horseshoe crab *Tachypleus tridentatus*,	Tachyplesin	Antifungal activity against *Candida albicans*	[61,62]
	Subphylum Crustacea (crabs, shrimps, and lobsters)	Crustins	Opsonization of bacteria	[115,116,117,118,119,120,121,122]
Vertebrates	Fish Japanese sea bass *Lateolabrax japonicus*	Hepcidine	Antimicrobial activity	[42,43,44,45,46,47]
	Orange-spotted grouper *Epinephelus coioides*	Epinecidin-1	Antifungal activity against *Botrytis cinerea* and gray mold on peach fruits	[31,32]
	Orange-spotted grouper *Epinephelus coioides*	Piscidin ecPis2S	Chemotactic and phagocytic activities, the respiratory burst of macrophages, mRNA expression of chemokine receptors, Toll-like receptors, T-cell receptors, and proinflammatory cytokines	[114,115]
	Hybrid striped bass *Morone saxatilis*	Piscidin-1	Anticancer activity: inhibits angiogenesis and induces apoptosis in oral squamous cell carcinoma through reactive oxygen species production	[142]
	Fish *Maurolicus muelleri* and *Benthosema glaciale*	QCPLHRPWAL	Anti-inflammatory and analgesic activities	[107]
	Carp *Cyprinus carpio*	KC14	Anti-inflammatory and anti-oxidant activities	[108]
	Chum salmon *Oncorhynchus keta*	MOP	Antioxidant activity	[143]

## Data Availability

Not applicable.
